# Prolonged Discontinuation Syndrome of Venlafaxine: A Case Study of Severe Gastrointestinal Manifestations

**DOI:** 10.7759/cureus.63560

**Published:** 2024-07-01

**Authors:** Khaled Alharmoodi, Janarth Kathirgamachelvam, Deena Nafri

**Affiliations:** 1 Liaison Psychiatry, The Lakes Mental Health Hospital, Colchester, GBR

**Keywords:** mirtazapine, gastrointestinal symptoms, depression, anxiety, serotonin discontinuation syndrome, discontinuation syndrome, venlafaxine

## Abstract

This case report describes a 26-year-old female with a history of childhood depression who experienced severe gastrointestinal symptoms and significant weight loss following the discontinuation of venlafaxine, a serotonin-norepinephrine reuptake inhibitor (SNRI). After tapering off the medication, days after cessation, she developed early satiety, nausea, bloating, and vomiting, leading to severe malnutrition with a body mass index (BMI) of 14. Despite the onset of symptoms being within the typical duration for discontinuation syndrome, extensive medical evaluations revealed no physical cause for her symptoms. Psychological assessment showed no current depression or anxiety, and she denied any eating disorder behaviors, suggesting a prolonged discontinuation syndrome. Her symptoms improved with the initiation of mirtazapine. This case underscores the importance of careful management when discontinuing venlafaxine, highlighting the potential for prolonged and severe discontinuation symptoms.

## Introduction

Venlafaxine, a serotonin-norepinephrine reuptake inhibitor (SNRI), is widely used to treat depression and anxiety disorders. Discontinuation syndrome associated with venlafaxine commonly manifests through a spectrum of symptoms. These include flu-like symptoms, characterized by malaise and possibly fever, along with sensations of dizziness, headaches, and nausea. Patients may also experience sensory disturbances, often described as "head zaps," in addition to insomnia and a sense of imbalance [1,2]. According to these sources, serotonin discontinuation syndrome typically lasts for a few weeks to a couple of months [1,2]. Rarely, symptoms may persist for longer periods, although it is unusual for the syndrome to endure for up to a year.

This case report details the experience of a 26-year-old British female with a history of childhood depression. Following the discontinuation of venlafaxine, she developed severe gastrointestinal (GI) symptoms and experienced significant weight loss. Despite undergoing comprehensive medical evaluations, which included gastroenterological investigations such as abdominal ultrasound, laboratory tests (complete blood count, comprehensive metabolic panel, thyroid function tests), and imaging studies (abdominal CT scan), no physical cause for her symptoms was identified. This absence of organic pathology suggests a potential diagnosis of prolonged discontinuation syndrome, where symptoms persist beyond the expected timeframe despite cessation of the antidepressant.

According to clinical guidelines, gradual tapering of venlafaxine is crucial to minimize the risk of severe discontinuation symptoms. Patients should be informed about the potential physical and psychological disturbances that can occur with abrupt discontinuation, as supported by various clinical studies and reports [3,4].

## Case presentation

A 26-year-old female with a history of childhood depression presented to the general hospital with a body mass index (BMI) of 14, indicative of severe malnutrition. She was admitted following a hypoglycemic episode, attributed to reduced food and fluid intake. This episode occurred against the backdrop of a nine-month history of progressively worsening GI symptoms. Additionally, she complained of intermittent headaches with tinnitus.

The patient has a longstanding history of depression and has been managed with venlafaxine for over five years. She reported being stable for years, which led her to attempt to wean off venlafaxine, originally prescribed at a dosage of 225 mg XL. She gradually reduced it to 75 mg without incident, lowering the dose every two weeks. However, further reduction to 37.5 mg led to adverse effects, including headaches, dizziness, and "head zaps." Subsequently, she discontinued venlafaxine entirely in August 2023 after careful titration.

Within a few days of cessation, the patient experienced the onset and escalation of GI symptoms. In August 2023, she experienced early satiety and reduced portion sizes. From September to October 2023, she developed nausea and bloating, occurring one to two hours postprandially. From November to December 2023, she consulted a general practitioner (GP), who prescribed increasing dosages of omeprazole, which proved ineffective. By January 2024, she found it increasingly difficult to ingest semi-solid and liquid foods, accompanied by vomiting episodes, predominantly consisting of undigested food, with no presence of blood or mucus.

Due to significant weight loss and a low BMI, a multidisciplinary team, including internal medicine, gastroenterology, and ENT (ear, nose, and throat) specialists, conducted comprehensive investigations to exclude medical etiologies. All diagnostic tests, including CT scans of the head and abdomen, an abdominal ultrasound, blood workup, and immunological assessments, returned normal results, revealing no clear physical cause for her symptoms.

Further exploration of the patient's mood and psychological state revealed no current evidence of depression or anxiety. The patient was unable to identify any additional stressors during this period. Despite the consideration of an eating disorder, she denied any problematic relationship with food. Specifically, she reported no preoccupation with her weight, no desire to achieve a thinner physique, no fear of weight gain, and no anxiety about eating (e.g., fear of choking or swallowing difficulties). She also denied being a picky eater or being triggered by the texture of certain foods. Moreover, she reported no compensatory behaviors such as excessive exercise, purging, or the use of laxatives, diuretics, or appetite suppressants. Mirtazapine was initiated at a dose of 15 mg, and the patient improved, starting to eat normally.

## Discussion

Venlafaxine, an SNRI, is widely prescribed for depression and anxiety disorders, but its discontinuation can lead to a spectrum of symptoms known as serotonin discontinuation syndrome. This case illustrates a prolonged presentation of such a syndrome in a 26-year-old woman following venlafaxine cessation. Common symptoms typically include dizziness, headache, nausea, and sensory disturbances, commonly referred to as "head zaps" [1,2]. While such symptoms usually resolve within weeks to a few months, they can persist longer, albeit rarely exceeding a year [2].

In this case, within days of completely stopping the medication, which is typical for the onset of serotonin discontinuation syndrome, the patient developed severe GI symptoms and experienced significant weight loss. Despite extensive medical evaluations ruling out organic causes and the absence of other psychiatric disorders that could potentially explain the presentation, these findings suggest a prolonged discontinuation syndrome, which may occur particularly after extended high-dose exposure to the medication.

The decision to initiate mirtazapine was based on its pharmacological properties. Mirtazapine, a noradrenergic and specific serotonergic antidepressant (NaSSA), acts as an antagonist at 5-HT3 receptors, which can alleviate GI symptoms such as nausea and vomiting. Moreover, its enhancement of serotonin and norepinephrine release via central alpha-2-adrenergic receptor blockade can mitigate symptoms of serotonin discontinuation syndrome [5]. The observed improvement with mirtazapine underscores its utility in managing prolonged discontinuation symptoms, despite it not being a selective serotonin reuptake inhibitor (SSRI). This highlights the broader applicability of NaSSAs in managing complex cases of antidepressant discontinuation syndrome, where conventional SSRIs or SNRIs may not suffice.

Discontinuation syndrome, particularly with venlafaxine, can be challenging due to its short half-life and potent impact on neurotransmitter systems. Abrupt cessation or inadequate tapering can lead to severe withdrawal symptoms, as evidenced in this case. Effective management strategies include adhering to structured tapering protocols recommended in clinical guidelines. These protocols advocate for gradual dose reduction over weeks to months, tailored to individual patient needs and treatment duration. Close monitoring and proactive symptom management are crucial to mitigate withdrawal effects and ensure patient safety during antidepressant discontinuation [3]. In some cases, such as ours, a slower reduction than suggested by standard protocols may be necessary to manage severe symptoms effectively.

## Conclusions

This case underscores the importance of careful management when discontinuing medications such as venlafaxine. Prolonged discontinuation syndrome, though rare, can present with severe and debilitating symptoms, such as the GI issues observed in this patient. Clinicians should be vigilant for atypical presentations of discontinuation syndrome and consider a gradual tapering schedule and close monitoring. Effective management strategies include adhering to structured tapering protocols and being prepared to adjust the tapering pace. In some cases, such as ours, a slower reduction than suggested by standard protocols may be necessary to manage severe symptoms effectively. Further research is needed to understand the mechanisms underlying prolonged discontinuation syndrome and to develop effective management strategies for affected patients.

## References

[1] Haddad PM, Anderson IM (2007). Recognising and managing antidepressant discontinuation symptoms. Adv Psychiatr Treat.

[2] Warner CH, Bobo WV (2023). Antidepressant discontinuation syndrome. StatPearls [Internet].

[3] Karasu B, Gelenberg A, Wang P (2000). Practice guideline for the treatment of patients with major depressive disorder. Am J Psychiatry.

[4] (2018). Clinical practice guidelines for mood disorders. https://www.ranzcp.org/files/resources/college_statements/clinical_guidelines/clinical-practice-guidelines-for-mood-disorders.aspx.

[5] Dew RE, Daniel SS, Goldston DB (2010). A prospective study of religion/spirituality and depressive symptoms among adolescent psychiatric patients. J Affect Disord.

